# Kazinol Q from *Broussonetia kazinoki* Enhances Cell Death Induced by Cu(ll) through Increased Reactive Oxygen Species

**DOI:** 10.3390/molecules16043212

**Published:** 2011-04-15

**Authors:** Bai-Luh Wei, Ying-Chieh Chen, Hsue-Yin Hsu

**Affiliations:** 1 Department of Life Science, National Taitung University, Taitung 950, Taiwan;Email: lavender07318307@yahoo.com.tw (Y.-C.C.); 2 Department of Life Science, Tzu-Chi University, Hualien 970, Taiwan; Email: hsueyin@mail.tcu.edu.tw (H.-Y.H.)

**Keywords:** kazinol Q, copper, DNA breakage, ROS

## Abstract

The ability of the flavan kazinol Q (**KQ**) to induce DNA breakage in the presence of Cu(II) was examined by agarose gel electrophoresis using supercoiled plasmid DNA. In **KQ**-mediated DNA breakage reaction, the involvement of reactive oxygen species (ROS), H_2_O_2_ and O_2_^-^ was established by the inhibition of DNA breakage by catalase and revealed DNA breakage by superoxide dismutase (SOD). The cell viability of gastric carcinoma SCM-1 cells treated with various concentrations of **KQ** was significantly decreased by cotreatment with Cu(II). Treatment of SCM-1 cells with 300 μM Cu(II) enhanced the necrosis induced by 100 μM **KQ**. Treatment of SCM-1 cells with 100 μM **KQ** in the presence of 300 μM Cu(II) increased the generation of H_2_O_2_. Taken together, the above finding suggested that **KQ** cotreatment with Cu(II) produced increased amounts of H_2_O_2_, thus enhancing subsequent cell death due to necrosis.

## 1. Introduction

Several kinds of natural products have subsequently been reported to act as DNA strand scission agents, including flavonoids, aurone, 5-alkylresorcinol, pterocarpanoids, biphenyl, stilbene, anthrapyrone, enediyne, macrocyclic lactams and lignoids that cleave DNA in the absence or presence of certain metal ions [1]. Many chemotherapeutic agents may be selectively toxic to tumor cells by producing an excess of Reactive Oxygen Species (ROS). Cytotoxic ROS signaling appears to be triggered by the activation of the mitochondrial-dependent cell death pathway through activation of the mitogen-activated protein kinase (MAPK) pathways and the proapoptotic Bcl-2 proteins, Bax or Bak, with subsequent mitochondrial membrane permeabilization and cell death [2]. Recently we reported that kaempferol-3-*O*-*β*-D-glucopyranoside with prooxidant activity at a higher concentration may mediate through the suppression of xanthine oxidase activity and reduce ROS induced by high concentrations of Cu(II) (500 μM) and prevent the subsequent cell death [3].

**Figure 1:**
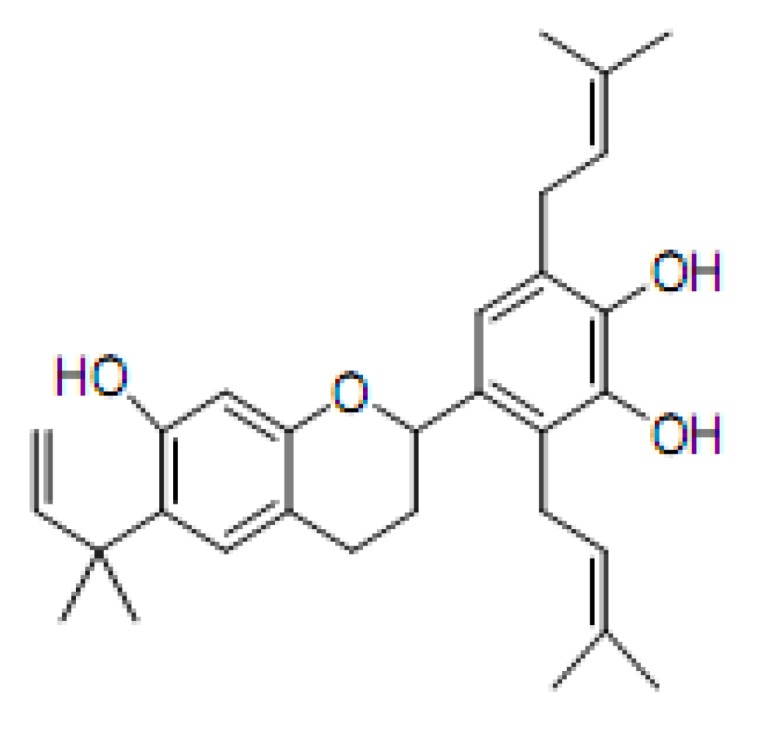
Structure of **KQ**.

Flavans, a large group of naturally occurring compounds, possess the basic flavonoid skeleton. The isolation and cytotoxicity of a new prenylflavan, kazinol Q (**KQ**) and two known prenylated 1,3-diphenylpropone derivatives, kazinols D and K from *Broussonetia kazinoki*, a Chinese crude drug, have been reported [4]. In continuation of our evaluation of the prooxidant activity of natural products in the present of Cu(II), we investigated the prooxidant activity of the abovementioned naturally occurring compound, **KQ** (Figure 1) and the mechanism of **KQ**-enhanced cytotoxicity induced by Cu(II) in SCM-1 cells.

## 2. Results

### 2.1. DNA Strand-Scission of Compound

**Figure 2:**
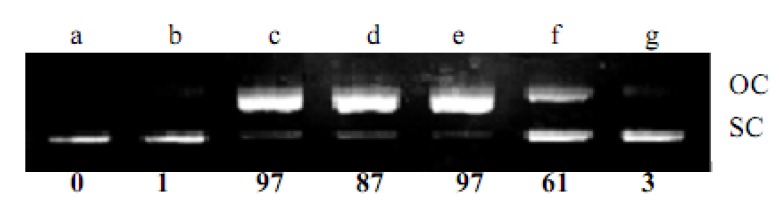
DNA strand scission by **KQ**. pBR322 plasmid DNA (500 ng) was incubated for 30 min at 37 °C in the presence of following additives: **(a)** 300 μM Cu(II); **(b)** 300 μM **KQ** ; **(c)** 300 μM **KQ +** 300 μM Cu(II); **(d)** 200 μM **KQ +** 200 μM Cu(II); **(e)** 100 μM **KQ +** 100 μM Cu(II); **(f)** 50 μM **KQ +** 50 μM Cu(II); **(g)** 25 μM **KQ +** 25 μM Cu(II).OC, open circular DNA; SC, supercoiled DNA.The percentage of OC DNA as compared to total DNA is individually shown below each lane.

**Figure 3:**
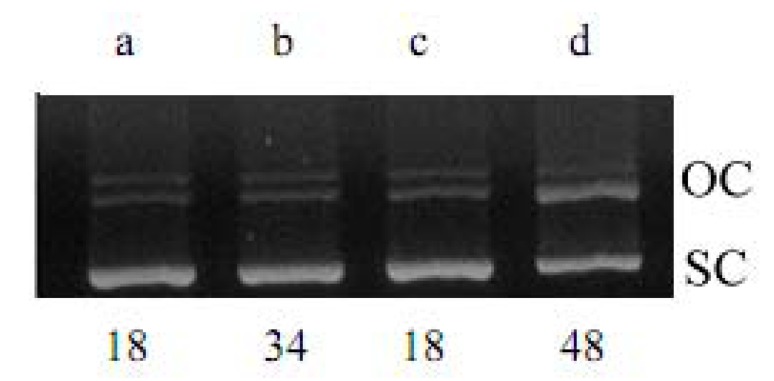
Effect of neocuproine (500 μM) on **KQ**-Cu(II) (300 μM) induced pBR322 DNA breakage. 500 ng pBR322 DNA was used in each lane. **(a)** pBR322 DNA; **(b)** DNA + Cu(II); **(c)** DNA + Cu(II) + neocuproine; **(d)** DNA + Cu(II) + **KQ** (100 μM) + neocuproine. OC, open circular DNA; SC, supercoiled DNA. The percentage of OC DNA as compared to total DNA is individually shown below each lane.

**Figure 4:**
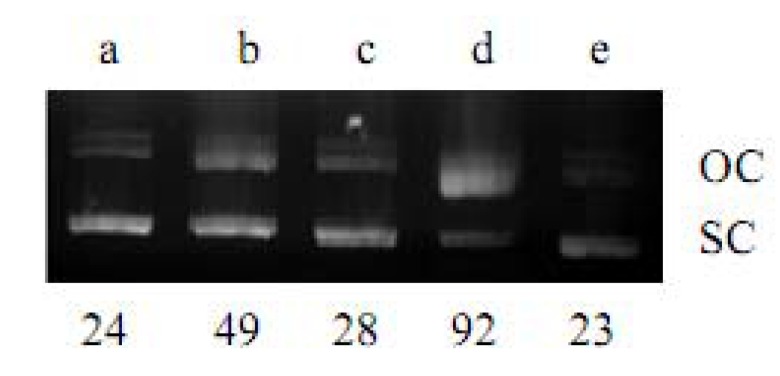
Effect of active oxygen scavengers on **KQ**-Cu(II)-mediated DNA breakage: **(a)** DNA alone; **(b)** DNA + Cu(II) (300 μM) + **KQ** (300 μM); **(c-e)** same as (b) with KI (750 μM), SOD (0.1 mg/mL), Catalase (0.1 mg/mL), respectively. OC, open circular DNA; SC, supercoiled DNA. The percentage of OC DNA as compared to total DNA is individually shown below each lane.

**KQ** was tested for its ability to convert supercoiled plasmid pBR322 DNA to relaxed open circles in the presence of Cu(II). As shown in Figure 2-Figure 4, **KQ** caused a significant level of Cu(II)-mediated DNA breakage in a concentration-dependent manner. The conversion of supercoiled DNA to a relaxed form induced by **KQ** in the presence of Cu(II) as shown in Figure 3 was partially inhibited with neocuproine, a Cu(I)-specific sequestering agent [5]. The **KQ**-mediated DNA breakage reaction was further tested for inhibition by various oxygen radical scavengers. As shown in Figure 4, **KQ-**Cu(II)-induced DNA degradation was inhibited by catalase and showed DNA breakage by KI and superoxide dismutase (SOD).

### 2.2. Cytotoxic Effect of KQ on SCM-1 Cells in the Absence or Presence of Cu(II)

**Figure 5:**
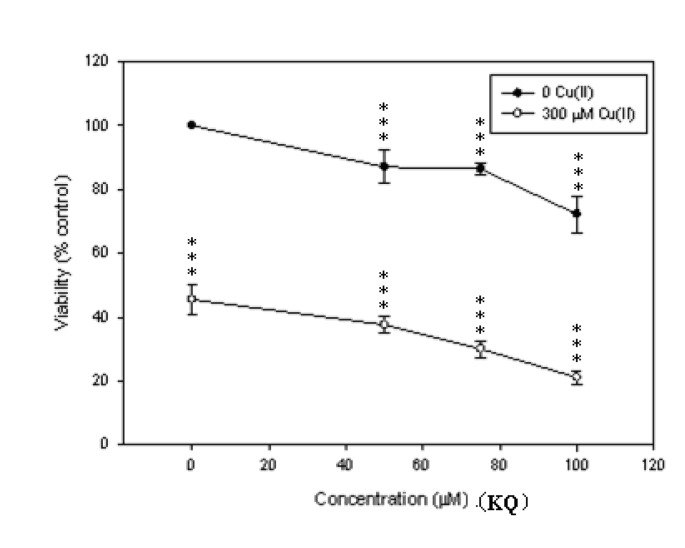
**KQ** potentiates cupric ion-induced SCM-1 cell death. Cell viability was assessed by the MTT assay 48 h after treating with different concentrations of **KQ** with or without Cu(II). *** *p < *0.001 represents significant differences compared with control values.

SCM-1 cells were treated with various concentrations of **KQ** for 48 h with or without Cu(II) and cell viabilities determined by the MTT assay (Figure 5). **KQ** caused increased cell death with increased concentrations at 50, 75, and 100 μM, respectively. 

**Figure 6:**
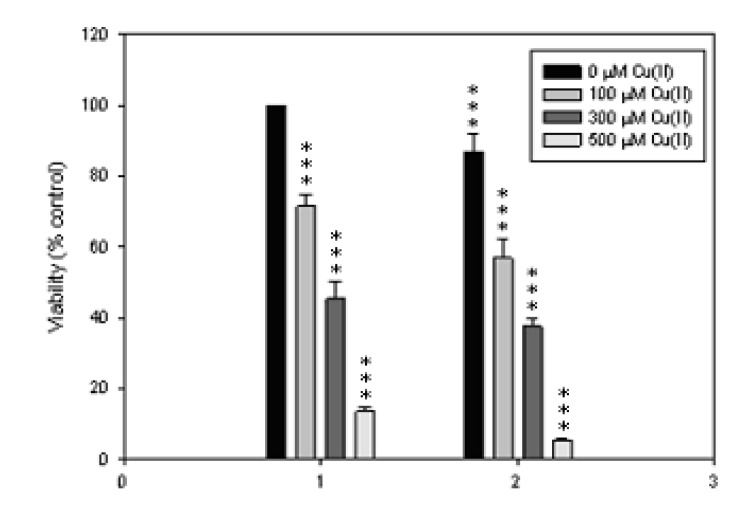
Copper-dependent increases in **KQ**-mediated cytotoxicity. SCM-1 cells were exposed to 0 (1) and 50 μM (2) **KQ**, respectively, and varying concentration of Cu(II) for 48 h and cell viability was measured by MTT assay. *** *p < *0.001 represents significant differences compared with control values.

Further evaluations showed that the cytotoxicity of **KQ** was potentiated by adding Cu(II) at a concentration of 300 μM. The cytotoxicity of either Cu(II) or 50 μM **KQ** against SCM-1 cells incubated for 48 h was enhanced with increasing concentrations of Cu(II) (Figure 6).

### 2.3. Mitochondrial Membrane Potential and Cell Apoptosis and Necrosis

**Figure 7:**
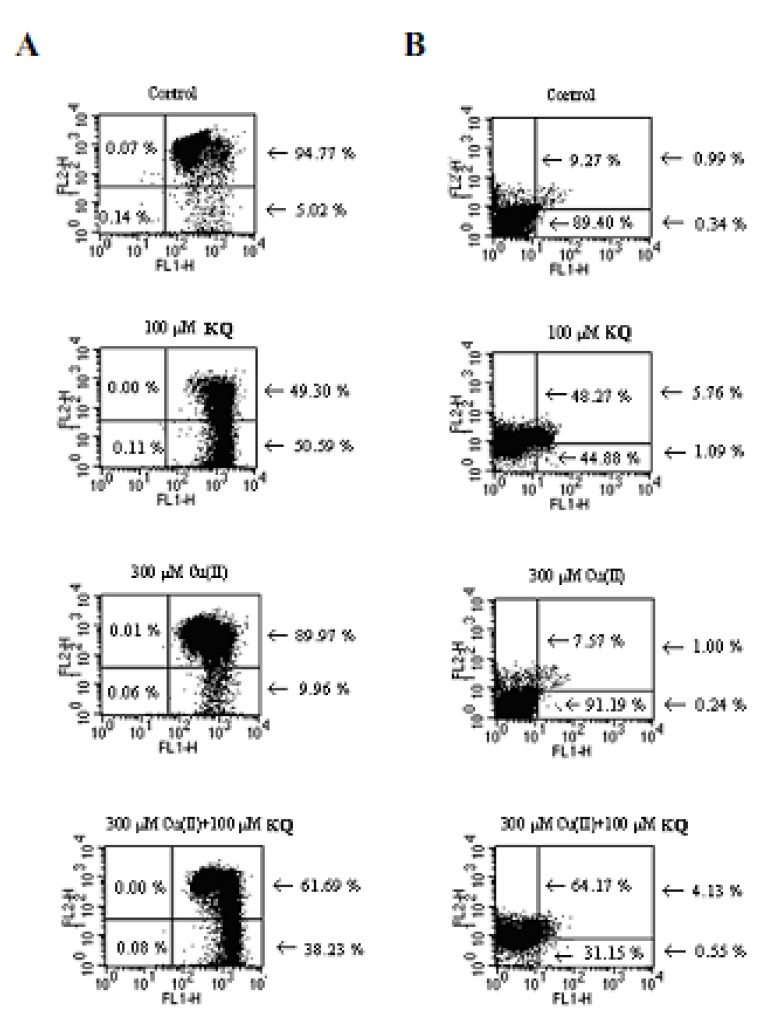
Effect of **KQ **on mitochondrial membrane potential with or without Cu(II). SCM-1 cells (1 × 10^4^) were treated with different concentrations of **KQ** with or without Cu(II) for 24 h. Cells were stained with JC-1 **(A)** and Annexin V/PI (lower quadrant: non-apoptotic cells, upper right: late apoptotic cells, lower right: apoptotic cells, upper left: necrotic cells) **(B)**, respectively, and analyzed by flow cytometry as described in methods. The control cells were treated with medium. The similar results were shown by three repeated experiments.

Treatment of SCM-1 cells with 100 μM **KQ** or 300 μM Cu(II) alone for 24 h produced significant change of △φm (Figure 7). Treatment of 300 μM Cu(II) and 100 μM **KQ** in SCM-1 cells for 24 h enhanced the change of △φm compared with that of SCM-1 cells treated with 300 μM Cu(II) alone (Figure 7). After treatment of SCM-1 cells with 100 μM **KQ** alone for 24 h, the cell numbers of apoptosis as well as necrosis were increased significantly, while treatment of SCM-1 cells with 300 μM Cu(II) alone did not increase the cell numbers of apoptosis and necrosis (Figure 7). When 300 μM Cu(II) treated SCM-1 cells were cotreated with 100 μM **KQ** for 24 h this significantly enhanced the necrosis cell number compared with those of treatment of SCM-1 cells with 300 μM Cu(II) alone or 100 μM **KQ **alone, while the apoptosis cell number of SCM-1 cells induced by 300 μM Cu(II) in the presence of 100 μM **KQ **was attenuated. 

### 2.4. Compound KQ Increased ROS Production with and without Cu(ll) Measured by Flow Cytometry

**Figure 8:**
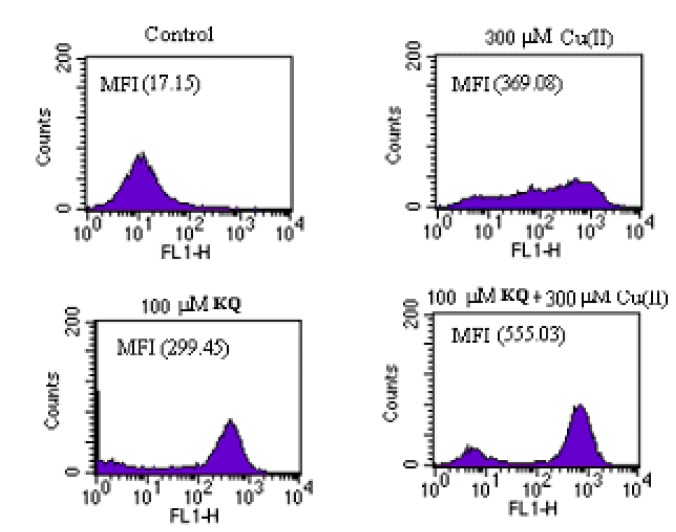
The Effect of **KQ** cotreated with or without Cu(II) on the production of H_2_O_2_. SCM-1 cells (1.5 × 10^4^) were treated with 300 μM Cu(II) or 100 μM **KQ **with or without 300 μM Cu(II), respectively, for 2 h and the amount of H_2_O_2 _was assayed by DCFDA staining. Each sample measured the mean fluorescence intensity (MFI) of 1.5 × 10^4^ cells corrected for autofluorescence. The control cells were treated with medium. Similar results were shown by three repeated experiments.

Compound **KQ **cotreatment with 300 μM Cu(II) or **KQ** alone showed increased SCM-1 cell death due to necrosis. We therefore hypothesized that **KQ** may affect cellular ROS generation. To test this, we monitored ROS levels using a fluorescence probe, dichlorofluorescin diacetate (DCFDA), which is nonfluorescent until it is oxidized by ROS within the cell. Intracellular ROS generation in control, 300 μM Cu(II), 100 μM **KQ**, and 100 μM **KQ** combined with 300 μM Cu(II)-treated SCM-1 cells for 2 h were assessed by flow cytometry following staining with DCFDA (Figure 8). **KQ,** Cu(II), and **KQ** combined with Cu(II)-treated SCM-1 cells exhibited an increase in mean dichlorofluorescin (DCF) fluorescence when compared with those of control, respectively.

## 3. Discussion

**KQ** gave a significant level of Cu(II)-mediated DNA damage in a concentration-dependent manner, except for 100 μM **KQ** combined with 100 μM Cu(II), while **KQ** alone did not significantly induce DNA damage (Figure 2). This indicated that prenylflavans such as **KQ** possess prooxidant activity. As shown in Figure 3, the conversion of supercoiled DNA to relaxed form induced by **KQ** in the presence of Cu(II) was partially inhibited by neocuproine, suggesting that that Cu(II) is not an essential intermediate in **KQ**-mediated DNA breakage and indicating that **KQ**-mediated DNA damage was associated with generation of ROS.

The **KQ**-Cu(II)-induced DNA degradation was inhibited by catalase and revealed DNA damage by KI and SOD. This indicated that H_2_O_2_ and O-. 2appeared to be partially involved in **KQ**-Cu(II)-mediated DNA breakage reaction.

As shown in Figure 5 and Figure 6, SCM-1 cells treated with various concentrations of **KQ** in the presence of 300 μM Cu(II) significantly potentiated the cytotoxicity induced by Cu(II). The cytotoxicity of 50 μM **KQ** on SCM-1 cells was enhanced significantly by cotreatment with increasing concentrations of Cu(II). This suggested that **KQ **significantly potentiated the cytotoxicity induced by Cu(II). Cu(II) was reported to induce both necrotic and apoptotic cell death in trout hepatocytes [6]. Our present data also demonstrate that 300 μM Cu(II) and 100 μM **KQ** induce both necrotic and apoptotic cell death, respectively (Figure 7). However, 100 μM **KQ **cotreatment with 300 μM Cu(II)enhanced necrotic cell death induced by Cu(II) in the cells, revealing that **KQ** has a potentiating effect on Cu(II)-induced cell death in SCM-1 cells due to necrosis. Cu(II) was reported to have the effect on enhancement of ROS production and these oxygen radical species play an important role in the regulation of cell cytotoxicity [6]. The origin of the radicals generated was at least partly mitochondrial [6]. In this study, the mitochondria membrane potential was reduced by treating with 100 μM **KQ **or 300 μM Cu(II) alone, while treating with 100 μM **KQ **combined with 300 μM Cu(II) did not enhance the decrease of the mitochondria membrane potential induced by 300 μM Cu(II) (Figure 7). Recent investigations have shown that the main source of ROS generated in the presence of Cu were the lysosomes, whereas the mitochondria appeared not to be involved [7]. Based on the above result and the weak reduction of mitochondria membrane potential by treating the cells with Cu(II) alone, it could be suggested that enhancement of cell death induced by **KQ **combined with Cu(II) is mitochondria-independent. 

ROS induce programmed cell death or necrosis, induce or suppress the expression of many genes, and activate cell signaling cascades, such as those involving mitogen-activated protein kinase [8]. As shown in Figure 8, treatment of SCM-1 cells with 100 μM **KQ** in the presence of 300 μM Cu(II) enhanced the production of H_2_O_2_ induced by 300 μM Cu(II) alone. The above results indicate that 100 μM **KQ** alone or 100 μM **KQ** cotreatment with 300 μM Cu(II) could enhance oxidative damage and subsequently induced cell death through increased generation of H_2_O_2_. 

## 4. Experimental

### 4.1. Chemicals

Ethidium bromide, bromophenol blue, Trizma, superoxide dismutase (SOD), and phosphate buffer solution (pH 7.4) were purchased from Sigma Chemicals (St. Louis, MO, USA). EDTA, cupric chloride and glycerol were purchased from J. T. Baker (Mallinckrodt Baker, Inc., Phillipsburg, NJ, USA). Supercoiled pBR322 plasmid DNA was purchased from ABgene House (Epsom, Surrey, UK). **KQ** was isolated and identified as previous report [4].

### 4.2. DNA Strand-Scission Assay

Reaction mixtures (25 μL) contain containing 10 mM Tris-HCl buffer (pH 8.0), supercoiled pBR322 plasmid DNA (500 ng), compound **KQ** (dissolved in DMSO, with final DMSO concentration less than 5%), CuCl_2_ and different components as described in the figures. Neocuproine or divalent metal ions were included in some experiments. Each batch of experiments included one blank control (DNA alone) and one metal control (DNA + Cu^2+^). After being incubated at 37 °C for 30 min, the reaction mixture was treated with 30% glycerol-0.01% bromophenol blue (5 μL) and analyzed by electrophoresis in a 1.0% agarose gel containing 0.7 μg/mL ethidium bromide. The electrophoresis was carried out in TBE buffer (89 mM Tris, 89 mM boric acid and 2 mM EDTA, PH 8.3) at 110-120 V for 2-3 h. Following electrophoresis, the DNA was imaged by ethidium bromide fluorescence which was photographed under ultraviolet light [5,9].

### 4.3. Cell Culture and MTT Assay for Cell Viability/Proliferation

SCM-1 gastric cancer cells were maintained in RPMI 1640 medium supplemented with 10% fetal bovine serum (FBS), glutamine, penicillin and streptomycin. The cells were cultured at 37 °C in a humidified atmosphere containing 5% CO_2_. For evaluating the cytotoxic effect of **KQ** with or without Cu(II), 8×10^3^ SCM-1 cells were plated in each well of 96-well microplate with 100 μL medium and incubated for 24 h before various treatments. Each drug was dissolved in dimethyl sulfoxide and mixed with culture medium to the treated concentrations and was then added to the culture with or without Cu(II). The maximum concentration of DMSO added to the medium in this study was 0.01%. After incubating for 24 h, 1 mg/mL MTT (dimethylthiazolyltetrazolium bromide) solution (100 μL) was added to each well and incubated for another 4 h and then 20% SDS in 50% dimethyl formamide (100 μL) was added and the formed crystals were dissolved gently by pipetting several times slowly. A plate reader was used to measure the absorbance at 540 nm using a μQuant ^TM^ (BioTek, USA) for each well. Viability was expressed as a percentage to the viable cells compared with untreated cells.

### 4.4. Measurement of Mitochondrial Membrane Potential Depolarization

A unique fluorescent dye, 5,5′,6,6′-tetrachloro-1,1′,3,3′-tetraethylbenzimidazolylcarbocyanine iodide, commonly known as JC-1 (Sigma, St. Louis, MO, USA), was used to measure mitochondrial depolarization in SCM-1 cells after treatment with **KQ** with or without Cu(II). The JC-1 dye assay was used for determination of reduction in mitochondrial membrane potential during apoptosis [6]. After treating with **KQ** and Cu(II) for 24 h, cells were harvested by trypsinization, washed with PBS buffer and 1 × 10^6 ^cells were resuspended in PBS (1 mL) containing 15 μM JC-1 dye for 30 min at 37 °C in the dark. Stained cells were washed, resuspended in 500 μL PBS and used for immediate FACS analysis (LSR, BD Biosciences, San Jose, CA). At least one thousand cells from each treatment were analyzed in this study.

### 4.5. Annexin V/PI Staining

SCM cells were washed twice with PBS before being suspened in 1 × annexin V binding buffer at a concentration of 1 × 10^-6^ cells/mL. Cells were transferred to a culture tube and and annexin V/PI (BD Pharmingen, San Diego, CA) were added. After gentle vortex, the cells were incubated for 20 minutes at room temperature in the dark. After adding 1 × annexin V binding buffer (400 µL) to each tube, cells were analyzed by flow cytometry (LSR, BD Biosciences, San Jose, CA). 

### 4.6. Compound KQ Induced ROS Production with and without Cu(II) Measured by Flow Cytometry

The intracellular H_2_O_2_ concentration was determined by measuring the fluorescent intensity of DCFDA (2′,7′-dichlorodihydrofluorescein diacetate) (Invitrogen Molecular Probes, Eugene, OR, USA) fluorescence dye. DCFDA was deacetylated by nonspecific esterase and further oxidized to a fluorescent compound, DCF (2′,7′-dichlorofluorescein) by cellular peroxides. In this study, SCM-1 gastric cancer cells were maintained in RPMI 1640 medium supplemented with the other cell culture components as described above. Cells were incubated with the indicated dose of **KQ** cotreated with or without Cu(II) for 2 h. Cells were then washed with PBS and incubated with 100 μM DCFDA at 37 °C for 30 min and harvested by trypsin-EDTA after washing twice with PBS. Red fluorescence was detected using a LSR flow cytometer (Becton Dickinson). Ten thousand events were evaluated for each sample. H_2_O_2_ production was expressed as mean fluorescence intensity (MFI) which was calculated by CellQuest software.

### 4.7. Statistical Analysis

Data were expressed as means ± S. D. Statistical analyses were performed using the Bonferroni *t*-test method after ANOVA for multigroup comparison and the student's *t*-test method for two-group comparison. *P* < 0.05 was considered to be statistically significant.

## 5. Conclusions

**KQ** or **KQ** cotreatment with Cu(II) induced oxidative stress or significantly enhanced oxidative stress induced by Cu(II) through increased the generation of H_2_O_2_ and this in turn caused apoptotic and necrotic cell death or necrotic cell death. Our findings suggested that **KQ** or **KQ** cotreatment with Cu(II) may have value in the treatment or prevention of certain cancers associated with decrease of H_2_O_2_.
